# Phase I study of nivolumab combined with IFN-β for patients with advanced melanoma

**DOI:** 10.18632/oncotarget.17090

**Published:** 2017-04-13

**Authors:** Taku Fujimura, Takanori Hidaka, Yumi Kambayashi, Sadanori Furudate, Aya Kakizaki, Hisayuki Tono, Akira Tsukada, Takahiro Haga, Akira Hashimoto, Ryo Morimoto, Takuhiro Yamaguchi, Tadao Takano, Setsuya Aiba

**Affiliations:** ^1^ Department of Dermatology, Tohoku University Graduate School of Medicine, Sendai, Japan; ^2^ Division of Nephrology, Endocrinology and Vascular Medicine, Department of Medicine, Tohoku University Hospital, Sendai, Japan; ^3^ Division of Biostatistics, Tohoku University Graduate School of Medicine, Sendai, Japan; ^4^ Clinical Research, Innovation and Education Center, Tohoku University Hospital, Sendai, Japan

**Keywords:** IFN-β, PD-1, safe dose, traditional rule-based 3 + 3 design

## Abstract

The efficacy of nivolumab is greater than that of other anti-melanoma drugs, so nivolumab-based combined therapies that enhance anti-tumor immune responses in patients with metastatic melanoma are of great interest to dermato-oncologists. As we have previously reported, IFN-β enhances the anti-tumor immune response of anti-PD-1 antibodies against B16F10 melanoma *in vivo*. To explore the potential of this property of IFN-β as part of a combination therapy for the treatment of metastatic melanoma patients, we performed a phase 1 trial, using a traditional rule-based 3 + 3 design, on patients with advanced melanoma. The nivolumab dose was fixed at 2 mg/kg, every 3 weeks. IFN-β was administered to three groups at doses of 1 million, 2 million, and 3 million units, respectively. Dose-limiting toxicities were defined as any grade 3-5 adverse events occurring between day 0 and day 42 that might possibly be related to nivolumab and IFN-β. Of the nine patients who received this combined therapy, none experienced dose-limiting toxicities, and all completed the treatment phase of the study. Patient follow-up continued for 6 months following the final treatment. There were two complete responses (22%) and one partial response (11%), all of which occurred in patients who had received monthly IFN-β immediately prior to the study. In this study, we determined the safe dose of IFN-β, when combined with nivolumab, to be 3 million units. To determine the efficacy of this combination therapy, further phase II trials are required.

## INTRODUCTION

The PD-1/PD-L1 pathway plays a critical role in tumor immune response, so nivolumab, an IgG4 anti-PD-1 antibody, is widely used in the treatment of various cancers, including advanced melanoma [1-3]. Nivolumab significantly prolongs survival in patients with metastatic melanoma, but only 31∼43% of patients who receive nivolumab monotherapy experience objective tumor regression [2, 3] Therefore, reagents that enhance the antitumor immune response induced by nivolumab are necessary to further optimize its use for the treatment of advanced melanoma. Recently, Larkin et al. reported that ipilimumab could significantly enhance the anti-melanoma immune response in advanced melanoma patients [2]. Administration of nivolumab in combination with local therapy [4-7], such as radiotherapy or contact immunotherapy, may also lead to improved outcomes. Although recent studies suggest that such nivolumab-based combined therapy might prove effective for treatment of advanced melanoma, the associated risk of immune-related adverse events (irAE), such as severe hepatitis, interstitial pneumonia, colitis, type 1 diabetes mellitus, hypophysitis, or myasthenia gravis, is an important consideration.

Interferon beta (IFN-β) has been used clinically as an adjuvant therapy for the treatment of melanoma, especially in Japan [8-9]. We previously reported that IFN-β enhances the anti-melanoma effects of anti-PD-1 Abs in mouse B16F10 melanoma by recruiting effector cells, instead of regulatory T cells (Tregs), to tumor sites *in vivo* [10]. In melanoma patients, peritumoral injection of IFN-β also recruits effector cells, including CD8 and TIA1-positive cytotoxic T cells (CTLs), into the tumor microenvironment [11], which suggests a possible mechanism for the therapeutic effects of IFN-β in the treatment of melanoma. In humans, IFN-β modulates the profiles of tumor-associated macrophages (TAMs) from M2 to M1 phenotypes, leading to a decreased proportion of Tregs among tumor infiltrating leukocytes (TILs) at the tumor site [10]. The major population of TAMs is composed of CD163^+^ M2 polarized macrophages [12], and anti-tumor agents (e.g., IFN-α, IFN-β, or IFN-γ) could activate TAMs [10, 13], which, once activated, could increase serum soluble (s)CD163 [14] and release various autoimmune related chemokines such as CXCL5 [15, 16]. Increased serum levels of sCD163 and CXCL5 correlate not only with autoimmune diseases such as atherosclerosis and rheumatoid arthritis [15-19], but also with adverse events in melanoma patients treated with nivolumab [20].

In this study, we performed a phase I trial, using the traditional rule-based 3 + 3 design, of combined nivolumab/IFN-β to determine a safe dose of IFN-β. In addition, we evaluated the efficacy of this combination therapy for advanced melanoma. The earliest that we expected severe nivolumab-induced irAEs (e.g., colitis, skin rash) might occur was 5∼6 weeks after initial administration [29], so we measured serum levels of sCD163 and CXCL5 (predictors of irAEs) at day 0 (to establish a baseline immediately before the administration of nivolumab and IFN-β) and at day 42, six weeks after the administration of nivolumab/IFN-β.

## RESULTS

### Patients

Nine patients were treated at Tohoku University Hospital, Sendai, Japan between January and October of 2016. All patients had received prior treatment. Patient characteristics are listed in Table 1.

**Table 1:** Patient characteristics (n=9)

		Age	Sex	Stage	Pretreatment	PS	primary sites
	Case 1	67	M	pT3bN3M1b stage IV	monthly IFNβ	ECOG 0	sole
IFN-β 1MIU	Case 2	68	M	pT4aN3M1b stage IV	monthly IFNβ	ECOG 0	sole
	Case 3	83	F	pT3bN2aM1a stage IV	Nivolumab	ECOG 1	sole
	Case 4	93	M	pT4bN0M1a stage IV	contact immunetherapy	ECOG 1	sole
IFN-β 2MIU	Case 5	74	M	pT2bN0M1b stage IV	monthly IFNβ	ECOG 0	upper arm
	Case 6	84	F	pT4aN3M1a stage IV	Nivolumab	ECOG 1	back
	Case 7	74	M	pT4aN1M1b stage IV	chemotherapy	ECOG 0	lower leg
IFN-β 3MIU	Case 8	58	F	pT4aN3M1c stage IV	chemotherapy	ECOG 0	lower lip
	Case 9	34	M	pT4aN3M1c stage IV	weekly IFN-α	ECOG 0	back

Performance States:PS

### Toxicities

Of the nine patients who received this combined therapy, none (0%) experienced dose-limiting toxicities (DLT), and all completed the treatment phase of the study. Patient follow-up evaluations occurred for 6 months following the final treatment. Three patients (33%) developed grade 1 or grade 2 AE (95% CI: 0%-66%). No patient in the study or follow-up phase (0%) developed grade 3 - 5 adverse events (AE). During the 6-month follow-up after the treatment period, six patients remained free of additional irAE. Two patients (cases 4 and 6) elected supportive care only during the treatment period and were lost to follow-up. One patient (case 8) developed grade 2 colitis during the treatment period, nine weeks after ipilimumab administration. Treatments were well tolerated and toxicities are summarized in Table 2.

**Table 2:** Patient demographic data, tumor stage, metastatic lesion status, immune-related adverse events, and tumor response.

		Pretreatment	Metastatic lesion	irAE	grade	Best response
	Case 1	monthly IFNβ	lung	ACTH deficiencies	2	irPR
IFN-β 1MIU	Case 2	monthly IFNβ	lung, pelvic LNs			irCR
	Case 3	nivolumab	pelvic LNs, in-transit			irPD
	Case 4	contact immunetherapy	pelvic LNs, in-transit			irPD
IFN-β 2MIU	Case 5	monthly IFNβ	lung			irCR
	Case 6	nivolumab	multiple in-transit			irPD
	Case 7	chemotherapy	lung, in-transit			irPD
IFN-β 3 MIU	Case 8	chemotherapy	lung, liver	fever	1	irPD
	Case 9	weekly IFN-α	bile duct	abdominal pain, fever	2	SD

PR: partial response; CR: complete response; PD: progression of disease; SD: stable disease

### Tumor response

There were two complete responses (irCR, 22%; 95% confidential intercal [CI]: 0%-44%), one partial response (irPR, 11%; 95% CI: 0%-22%), one patient whose disease remained stable (irSD, 11%; 95% CI: 0%-22%), and five patients who experienced disease progression (irPD, 55%; 95% CI: 0%-110%). All patients who received adjuvant monthly IFN-β before the metastasis of melanoma (case 1, case 2, and case 5) responded well to nivolumab with IFN-β. The best response was in case 9, who received weekly adjuvant IFN-α and achieved stable disease. Of the other patients in this study, those who had received chemotherapy, nivolumab, or contact immunotherapy prior to melanoma metastasis progressed to disease. Hence the objective response rate was 33% (95% CI: 0%-66%). Tumor responses of individual patients are listed in Table 2. Five of the nine patients (55%) experienced disease progression and changed treatment during follow-up.

### Serum levels of sCD163 and CXCL5

**Figure 1:**
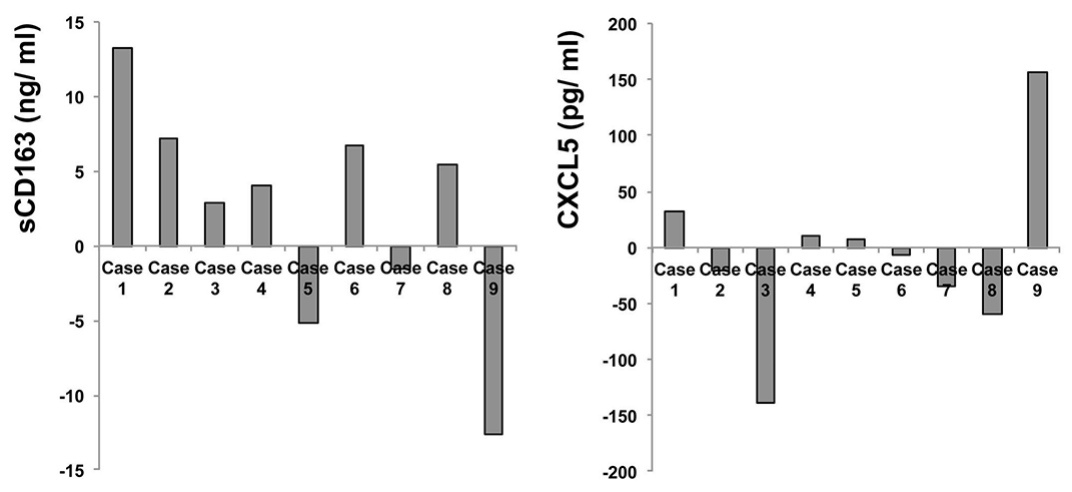
Serum levels of sCD163 and CXCL5 at days 0 and day 42 The serum levels of sCD163 **(A)** and CXCL5 **(B)** from each patient at day 0 and day 42 were examined by ELISA. These data represent changes of serum sCD163 and CXCL5 at day 42 compared to day 0.

Compared to baseline (day 0), serum levels of sCD163 and CXCL5 at day 42 were both prominently increased in case 1, a patient who developed grade 2 isolated ACTH deficiency. The serum level of sCD163 was prominently decreased and CXCL5 was increased in case 9, a patient who developed grade 2 abdominal pain and grade 1 fever. There were no remarkable changes in sCD163 and CXCL5 levels in the remaining seven patients (Figure 1).

## DISCUSSION

Because of nivolumab’s higher efficacy than other anti-melanoma drugs (e.g., ipilimumab and dacarbazine) [1, 21], and because it induces a longer duration of anti-tumor response than BRAF/MEK inhibitors (e.g., vemurafenib, dabrafenib, and trametinib) [22, 23], dermato-oncologists are particularly interested in combining nivolumab with agents that enhance the anti-tumor immune response in patients with metastatic melanoma [3, 10, 24]. The efficacy of nivolumab is significantly increased when combined with ipilimumab (57.7%), and while unfortunately the rate of severe treatment-related adverse events (grade 3 or 4) is also significantly increased with this particular combination (55.0%) [2], the findings suggest that the anti-tumor immune response induced by nivolumab could be increased by other immune systems. Because the anti-tumor effects of nivolumab are determined, at least in part, by the number of TILs and their expression of PD-1 [3], and because IFN-β increases the number of PD-1-expressing TILs at melanoma tumor cites, *in vivo* [10], we hypothesized that IFN-β could improve the efficacy of nivolumab for treatment of human metastatic melanoma. Full testing of this hypothesis will require a randomized controlled phase II trial, but first, the safe dose of IFN-β to be combined with the conventional dose of nivolumab (2 mg/kg) had to be determined. IFN-β recruits effecter T cells to tumor sites in both mice and humans [10, 11], which might be expected to hasten the development of treatment-related AEs. Here, using a traditional rule-based 3 + 3 design, we both determined the safe dose of IFN-β to be used in the combined therapy and demonstrated that there is no increased risk of severe treatment-related AEs associated with the combination of IFN-β with nivolumab, either during the six-week treatment period or during a six-month follow-up.

CXCL5 is a biomarker of Th17-mediated autoimmune diseases, such as multiple sclerosis, rheumatoid arthritis, and pemphigus vulgaris [15, 16, 25], and sCD163 is an activation marker for CD163^+^ TAMs that appears in the serum as a result of proteolytic shedding [17]. TAMs release CXCL5 via stimulation of periostin [26], and the tumor stroma of melanoma possesses prominent periostin [27, 28] and TAMs [8, 12], so the increased serum levels of sCD163 and CXCL5 in a patient who develops adverse nivolumab-induced, immune-related events are presumably related to periostin-stimulated TAM activation. In the setting of our combination therapy, therefore, sCD163 and CXCL5 serum levels could be valuable predictors of adverse events [20]. We therefore measured serum levels of sCD163 and CXCL5 at days 0 (immediately before the administration of nivolumab and IFN-β) and day 42, the earliest point at which severe irAEs (colitis, skin rash) caused by nivolumab might be expected to occur [29]. As mentioned above, the serum levels of sCD163 and CXCL5 were increased in case 1, a patient who developed grade 2 idiopathic ACTH deficiencies, though the increased level was not drastic compared to a previously-reported case [20] in which the patient developed grade 4 idiopathic ACTH deficiencies. The serum level of sCD163 was prominently decreased and CXCL5 was increased in case 9, a patient who developed grade 2 abdominal pain and grade 1 fever. These findings suggest that fluctuations in serum levels of sCD163 and CXCL5 might differ across the spectrum of possible adverse events. There was no remarkable change in the serum sCD163 and CXCL5 levels in patients who did not develop treatment-related adverse events.

In this 3 + 3 design phase I clinical trial, we determined that the safe dose of IFN-β in combination with nivolumab is 3 million units. In addition, we found that the rate of complete tumor response among patients in our study was 22.2% (95% CI: 0%-44.4%), which is higher than previously reported for nivolumab monotherapy (8.9%) [2], though admittedly, the number of patients in our study was very small. It should also be noted that the patients who exhibited the best responses had received monthly adjuvant IFN-β therapy prior to melanoma metastasis, so it is possible that pre-treatment with IFN-β might also enhance the anti-tumor effects of nivolumab. Overall, our results suggest that IFN-β does not increase the rate of immune-related adverse events, and that it might enhance the anti-melanoma effects of nivolumab.

## PATIENTS AND METHODS

Patients were eligible if they had unresectable stage III melanoma, if their tumor was resectable but they had declined resection, or if they had stage IV melanoma with accessible cutaneous, subcutaneous, and/or nodal lesions (patients were staged according to the AJCC Staging Manual, 7^th^ Edition, 2011). Other inclusion criteria were: age of at least 20 years; Eastern Cooperative Oncology Group (ECOG) performance status of 0 to 1; and adequate bone marrow and liver function. Exclusion criteria were: active autoimmune disease; history of hypersensitivity to nivolumab or IFN-β; interstitial pneumonia; cancer originating in other organs; psychological disorders; and concurrent therapy with any other anti-melanoma chemotherapeutic drugs.

### Study design and treatment

**Figure 2:**
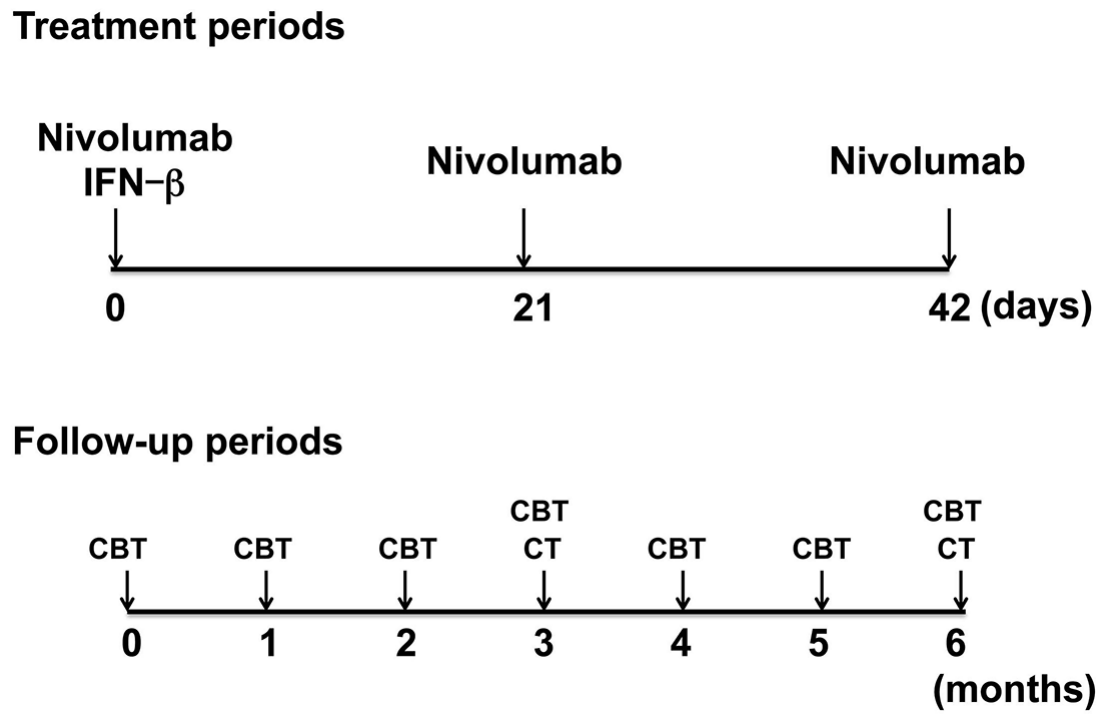
The time-line of events CBT: conventional blood test. CT: computed tomography.

This phase 1 trial (UMIN000020222) was an open label, non-randomized, traditional rule-based 3 + 3 design. The intravenous administration of nivolumab was fixed at 2 mg/kg every 3 weeks (Figure 2). IFN-β was intra-dermally administered at 1 million units, 2 million units, and 3 million units to the three patients in each group at the site of the primary tumor. We set the maximum tolerated dose at the conventional IFN-β therapeutic dose approved in Japan (3 million units).

### Assessment

All study patients were evaluated for DLT during the first 6 weeks of treatment. Adverse events were graded using NCI CTCAE version 4.03. DLT were defined as grade 3-5 adverse events at least possibly related to nivolumab and IFN-β that occurred between day 0 and day 42. The first three patients received nivolumab (2 mg/kg) and IFN-β (1 million units) at day 1 (dose level 1+) as per protocol design. With 0 of 3 patients exceeding a DLT of grade 3, three more patients were evaluated at dose level 2+ of nivolumab (2 mg/kg) and IFN-β (2 million units) at day 1. With no DLT reported in any of the three patients in level 2+, three more patients were evaluated at dose level 3+ of nivolumab (2 mg/kg) and IFN-β (3 million units) at day 1. With no DLT reported at level 3+, we finalized the study. After the treatment period, patients were assessed every 3 weeks with physical examination, conventional blood examination, and chest radiography, and assessed every 3 months with follow-up computed tomography (CT) scans. The tumor response was clinically evaluated by measuring the longest diameter of the target lesions over time. A partial response (irPR) was defined as a decrease of >30%, while progressive disease (irPD) was defined as an increase of >20%, as compared to the baseline measurement. Complete response (irCR) corresponded to the disappearance of all target lesions. We measured tumors 3 months and 6 months after the treatment period. Blood samples were obtained on day 0 (at the first administration of nivolumab and IFN-β), day 21 (second administration of nivolumab), and day 42 (third administration of nivolumab). We measured serum levels of sCD163 and CXCL5 at days 0 and 42.

### Study oversight

The study protocol and all amendments were approved by the institutional review board at Tohoku University Graduate School of Medicine (2016-2-023). The study was conducted in accordance with the Declaration of Helsinki and with Good Clinical Practice guidelines as defined by the International Conference on Harmonisation. All patients provided written informed consent before enrollment. A data and safety monitoring committee was established to provide oversight of safety and efficacy considerations. The study was registered with UMIN (UMIN000020222).

### ELISA

We analyzed day 0 and day 42 serum soluble (s) CD163 and CXCL5 levels by ELISA according to the manufacturer’s protocol (R&D Systems).

### Statistical methods

For each dose group, DLT and response rate and its 95% confidence interval were estimated.
